# Huachansu suppresses human bladder cancer cell growth through the Fas/Fasl and TNF- alpha/TNFR1 pathway *in vitro* and *in vivo*

**DOI:** 10.1186/s13046-015-0134-9

**Published:** 2015-02-25

**Authors:** Tao Yang, Runlin Shi, Lei Chang, Kun Tang, Ke Chen, Gan Yu, Yuanfeng Tian, Yonglian Guo, Wei He, Xiaodong Song, Hua Xu, Zhangqun Ye

**Affiliations:** Department and Institute of Urology, Tongji Hospital, Tongji Medical College, Huazhong University of Science and Technology, Wuhan, 430030 China; Department of Urology, Central Hospital of Wuhan, Wuhan, 430014 China

**Keywords:** Huachansu, Bladder cancer, Apoptosis, TNF- alpha/TNFR1, Fas/Fasl

## Abstract

**Background:**

Huachansu (HCS), a class of toxic steroids extracted from toad venom, which has been shown to be a valuable anticancer drug in many kinds of cancers. However, the effect of HCS on bladder cancer has not been elucidated. In this study, we focused on the antitumor activities and related mechanisms of HCS on bladder cancer *in vitro* and *in vivo*.

**Methods:**

Cell viability of T24, EJ, RT-4, SV-HUC-1 cells after HCS treatment was measured by MTS, whereas the changes of cell morphology were observed by transmission electron microscopy. The early apoptosis induced by HCS was evaluated by flow cytometry, and the expression level of apoptosis-related molecules (Bax, Bcl-2, XIAP, PARP, cleaved-Caspases 3, 8, 9) was detected using Western blot. We then evaluated the impact of HCS on the expression of Fas/Fasl, TNF- alpha/TNFR1, and the activation of NF-Kappa B pathway, and furthermore the effect of these pathways in HCS induced-apoptosis were also detected. At last, xenograft tumor in nude mice was used to further investigate the antitumor effect of HCS *in vivo*.

**Results:**

Our results showed that HCS could efficiently inhibit proliferation and induce apoptosis in human bladder cancer cell lines. The expression of Fas, Fasl, TNF- alpha were all elevated at both mRNA and protein level after HCS treatment. Furthermore, down regulation of TNF- alpha, TNFR1, Fas or inhibition of Fas/Fasl interaction decreased the relative number of death cells induced by HCS. *In vivo*, HCS treatment significantly suppressed tumor growth and induced apoptosis in xenografts tumor in nude mice.

**Conclusions:**

HCS could efficiently inhibit proliferation and induce apoptosis in human bladder cancer cells *in vitro* and *in vivo*, which is largely mediated by Fas/Fasl and TNF- alpha/TNFR1 pathway.

**Electronic supplementary material:**

The online version of this article (doi:10.1186/s13046-015-0134-9) contains supplementary material, which is available to authorized users.

## Background

Bladder cancer is a major public health problem worldwide. More than 90% of the bladder cancers are urothelial cell carcinomas (UCC) and approximately 70% of bladder tumor present as non-muscle-invasive bladder cancer (NMIBC) [1,2]. The standard treatment for patients with superficial bladder cancer is transurethral resection (TUR) of tumors. However, nearly 60% to 70% of these tumors will recur, and 25% will progress into a higher stage or grade [3,4]. Although many chemical agents have shown some evidence of activity against tumor recurrence, their toxicity and incomplete efficacy have limited their use as common chemotherapy agents [5]. These factors highlight the urgent of novel adjuvant agents. Natural products, including those from plants and microorganisms, provide much potential for anticancer drug discovery [6,7].

Chansu, the dried toad venom or the dried secretion from the skin glands of Bufo bufo gargarizans Cantor or B.melanotictus Schneider has long been used for cancer treatment in China and other Asian countries, such as the treatment for liver and pancreatic cancer [8-10]. Huachansu (HCS) is an injectable form of chansu, the extract contains several biologically active substances, primarily indole alkaloids (bufotenine, bufotenidine, and cinobufotenine) and steroidal cardiac glycosides (bufalin, resibufogenin, cinobufagin, cinobufotalin, marinobufagin, and bufotalin). Recent studies showed that the antitumor activity of HCS can be attributed mainly to the cardiac glycosides it contains, including bufalin, resibufogenin, and cinobufagin. High-pressure liquid chromatography (HPLC) analysis determined the relative concentrations of the components. Bufalin was present at the highest concentration, followed by moderate levels of cinobufagin [11]. Chansu extractions including Bufalin and cinobufagin induce apoptosis and inhibit proliferation of many cancer cells through different pathways, Bufalin induces apoptosis by activation of activator protein-1 (AP-1), the c-JunN-terminal protein kinase, Rac1, mitogen-activated proteinkinase [9,12] in human leukemia U937 cells, as well as by inhibition of phosphatidylinositol 3-kinase (PI3K)/Akt signaling in human gastric cancer cells [13]. Bufalin and cinobufagin induce apoptosis of human prostate cancer cells partialy with Fas stimulation, Bax translocation, cytochrome c release and caspase activation [14]. However, the effect of HCS on bladder cancer has not been elucidated. So in this study, we investigated the anticancer effects and related molecular mechanisms of HCS on bladder cancer *in vitro* and *in vivo*.

## Methods

### Cell culture and reagents

Bladder cancer cell lines T24 and EJ were obtained from the Institute of Biochemistry and Cell Biology, Shanghai Institutes for Biological Sciences, Chinese Academy of Sciences (Shanghai, China), and maintained in RPMI-1640 medium. SV-HUC-1 and RT-4 were also purchased from this place and maintained in DMEM/F-12 and McCoy's 5A medium respectively. Cells were cultured added with 10% fetal bovine serum (FBS) in a humidified atmosphere of 5% CO_2_ maintained at 37°C. HCS was obtained from Anhui Jinchan Biochemistry Company Ltd., in Huaibei, China (Chinese FDA (Z34020274)), PDTC was purchased from Sigma-Aldrich and ZB4 was bought from GeneTex.

### Cell viability assay

Briefly, T24, EJ, RT-4 and SV-HUC-1 cells (3 × 10^3^ per well) were plated in 96-well plates and incubated with various dilutions of standard HCS(1:400, 1:200, 1:100, 1:50) for 24, 48 and 72 h. Cell viability was then detected using CellTiter 96®AQueous Non-Radioactive Cell Proliferation Assay (MTS) (Promega, USA). Three independent experiments with triplicate were carried out.

### Transmission electron microscopy

T24 cells were pretreated with the indicated dilution of HCS(1:100) for 24 h and 48 h, respectively. The treated cells were then collected and fixed with 2.5% glutaraldehyde. Ultrastructure of the cells was examined on a transmission electron microscopy (Tecnai, Netherlands) at 2500× magnification.

### Flow cytometry

Cellular apoptosis was determined by annexin V-FITC/PI staining using flow cytometry. T24 and EJ cells were pretreated with different dilutions of HCS for 24 h. After that, cells (1 × 10^6^) were collected, centrifuged and washed with phosphate buffered saline(PBS) for two times. 300 μL Binding buffer was then added to each tube and cells were re-suspended. The supernatant cells were incubated with 5 μL of annexin V-FITC and 5 μL of PI for 15 min at room temperature in the dark. Then, the apoptotic analyses were done by flow cytometry within one hour.

### RNA extraction and quantitative real-time RT-PCR

Total RNA was extracted with the TRIzol reagent (Invitrogen Life Technologies). RNA yield and quality were determined with a Nano Drop 1000 Spectrophotometer. For cDNA synthesis, 1 μl of total RNA was reverse-transcribed using Toyobo RT reagent Kit (Perfect Real Time) according to the manufacturer’s instructions. Quantitative real-time RT-PCR was carried out using using SYBR Premix Ex Taq on MX3000 instrument. The PCR amplification program consisted of an initial polymerase activation at 95°C for 10 min, followed by 40 cycles at 95°C for 5 s, 60°C for 30 s and 72°C for 30 s for those for genes. The results were normalized with housekeeping gene Beta-actin. Sequences of primers are listed in Table 1.

**Table 1:** **Primer sequences for RT-PCR**

**Target**	**Upstream primer**	**Downstream primer**	**Accession number**
Fas	TCTGGTTCTTACGTCTGTTGC	CTGTGCAGTCCCTAGCTTTCC	NM_152872
Fasl	AACTCAAGGTCCATGCCTCTG	GGTGAGTTGAGGAGCTACAGACA	NM_000639
TNF-α	TGTAGCCCATGTTGTAGCAAA	CAAAGTAGACCTGCCCAGACT	NM_000594
TNFR1	TCCTTCACCGCTTCAGAAAA	GGGATAAAAGGCAAAGACCAA	NM_001065
β-actin	TCCTGACCCTGAAGTACCCCATTG	GGAACCGCTCATTGCCGATAGT	NM_001101

### Protein extraction and Western blot analysis

Cells after treated with HCS were harvested and lysed with lysis buffer (50 mM Tris (pH7.4), 50 mM NaCl, 1 mM EDTA, 1% Triton X-100, and 10% glycerol). All solutions contains protease inhibitor cocktail (Sigma) and mixture of phosphatase inhibitors (10 mM NaF, 1 mM sodium pyrophosphate, 1 mM sodium orthovanadate, and 0.1 mM β- glycerophosphate^)^, and the protein extraction was processed according to the manufacturer's instructions. Protein concentration was measured using a BCA Protein Assay Kit (Beyotime, Wuhan, China) according to the manufacturer's instructions. For Western blot analysis, an equal amounts of 50 μg protein were subjected to electrophoresis on SDS-polyacrylamide gels and transferred onto polyvinylidene fluoride (PVDF) transfer membranes by western blotting. Blots were probed with the following antibodies Bax, Bcl-2, XIAP, cleaved Caspase-3,-8,-9, cleaved PARP, p65, p-p65, iκB-α (Cell Signaling Technology, USA), Fas, Fasl, (epitomics,USA) and TNFR1 (Proteintech group,USA ) overnight at 4°C. Then followed by secondary antibody-conjugated horseradish peroxidase (HRP) and detected by ECL solution .

### ELISA assay for TNF-α secretion

After cells were treated with HCS for 4 h and 8 h, the level of TNF-α protein accumulating in the medium were determined using an TNF-α ELISA kit (ExCell Biology, Shanghai, China). The assay was performed in duplicate according to the manufacturer’s recommendations.

### siRNA trasfection

siRNA targeted against Fas (5’- GUGCAAGUGCAAACCAGACTT -3’), TNFR1 (5’- CAAAGGAACCUACUUGUACUU -3’), TNF-a (5’- GUGCUGGCAACCACUAAGA -3’;) or control siRNA (5’-CCCCUUUUAAAAGGGGCCC-3’) was transfected into T24 and EJ cells using Lipofectamine RNAiMAX (Invitrogen) to a final concentration of 50 pmol/ml according to the manufacturer’s recommendations. 48 hours after transfection, knockdown was assessed by PCR from a parallel transfection.

### Bladder cancer xenograft model and *in vivo* therapy with HCS

All animal procedures were carried out with the approval of the Animal Ethics Committee of the Huazhong University of Science and Technology. 4-week old female athymic nude mice (BALB/c-nu/nu mice) were inoculated subcutaneously with 5 × 10^6^ T24 cells. After 7 days, 98% of mice grew visible tumors. The mice were randomized and assigned to the control group or the experimental group. Mice in the control group were percutaneously injected with 0.4 ml saline daily, and mice in the experimental group were percutaneously injected with 0.4 ml HCS(1:2, 1:1) into the tumor every day. The tumors were measured twice a week with microcalipers, and the tumor volumes were calculated using the following equation: Tumor volume(mm^3^) = 1/2× (tumor length) × (tumor width)^2^. And the body weight of the mice was recorded twice a week. At the end of experiment, tumors were excised, weighed, and then each tumors were fixed in 4% of paraformaldehyde for further analyses.

### In situ apoptosis detection by TUNEL staining

After desired treatment, the paraffin-embedded sections of samples were studied by terminal deoxynucleotidyl transferase-mediated dUTP nick-end-labeling (TUNEL) assay. Staining was carried out according to the protocol provided by the supplier. Apoptosis was evaluated by counting the positive cells as well as the total number of cells at 10 arbitrarily selected fields at 400× magnification in a blinded manner.

### Immunohistochemistry staining

Immunohistochemistry (IHC) was conducted as previously described [15]. Tissues were deparaffinized, rehydrated, and incubated at room temperature in 0.3% H_2_O_2_ to block endogenous peroxidase and in blocking solution for nonspecific binding. Primary antibody were applied to sections overnight at 4°C. Afterwards, tissues were incubated with anti-mouse HRP conjugated (Abcam, USA) secondary antibody for 1 h at room temperature. Then enzyme development was performed with DAB/H_2_O_2_ complex for 10 min at room temperature and in the absence of light which provides a brownish precipitation. The primary antibodies specific for Fas, Fasl (epitomics, USA) ,TNF-α and TNFR1(Proteintech group,USA) were used at working dilution 1:50, 1:100, 1:50 and 1:200 respectively. Stained (brown) cells were quantified as number of positive cells. To evaluate the intensity of antigen immunoreactivity we examined the percent of positive staining urothelial cells in 10 fields at random per rat per antibody under 400 × magnification.

### Statistical analysis

Statistical analysis was performed using the software of Statistical Package for the Social Sciences Version 16 for Windows. Data from 3 to 5 independent experiments were calculated as means and standard deviations. Comparisons of results between treated and control groups were made by the Student t tests. A P-value of less than 0.05 was considered significant.

## Results

### HCS inhibits the viability of human bladder cancer cells

**Figure 1:**
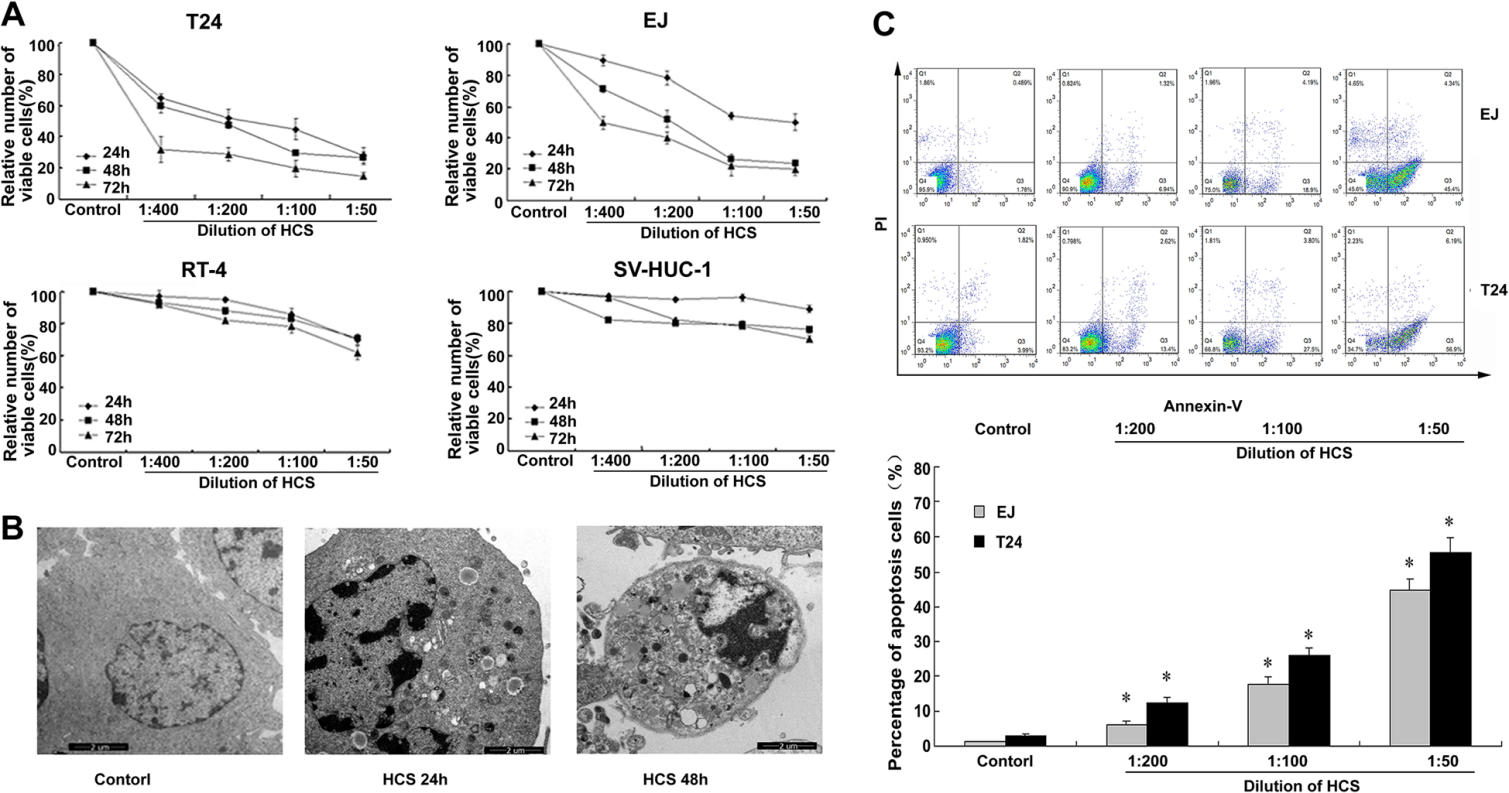
**HCS prohibited bladder cancer cells growth and induced apoptosis of T24 and EJ cells. (A)** Effect of HCS on proliferation of T24, EJ, RT-4, and SV-HUC-1 cells. Values are given as a percentage of untreated control cells. The data are presented as the average for triplet results from a representative experiment; bars, SD. **(B)** The ultrastructural morphologic changes of the T24 cells treated with HCS (1:100) under the transmission electron microscope. **(C)** The apoptotic fraction of cells detected by annexin V staining (x-axis)/propidium iodide staining (y-axis) after HCS treatments and the percentages showing the annexin V-positive/PI-negative fraction. Columns are expressed as mean ± SD of 3 independent experiments. *, *p* < 0.05 for HCS vs. control.

After cells were treated with various dilutions of HCS for 24, 48 and 72 h, cell viability of T24 and EJ, measured by the MTS assay, decreased significantly in a dose- and time-dependent manner (Figure 1A). Meanwhile, HCS showed little inhibition effect on less malignant RT-4 cells and immortalized SV-HUC-1 cells. This phenomenon indicated that HCS may have less damage on normal bladder tissue.

### Morphologic change induced by HCS

To get some detailed morphologic changes, we used transmission electron microscopy. As shown in Figure 1B, The normal cells (normal saline) showed untreated cells with intact nuclear membrane, huge and circular nuclei, more chromatin, abundant mitochondria and endoplasmic reticulum with good morphology. Compared with the control, the cells treated with HCS for 24 h exhibited that the chromatin accumulation inside the nuclear membrane lumped, a large number of autophagocytic vacuoles formed, and the mitochondria were damaged. After cells were incubated for 48 h, the cells became smaller, organelles were destroyed, partial nuclear membranes were disrupted and nuclei broke up.

### Apoptosis effect of HCS on bladder cancer cells

The apoptotic effect of HCS on bladder cancer cells was detected through Annexin V-FITC/PI double staining assay. Results demonstrated that HCS had an effect on increasing apoptosis. With Annexin V staining, early apoptosis was clearly detectable in the two bladder cancer cells treated with HCS. Compared to the control group, the cell apoptotic rates were significantly increased in a dose-dependent manner (Figure 1C).

### The effect of HCS on apoptosis-related molecules

**Figure 2:**
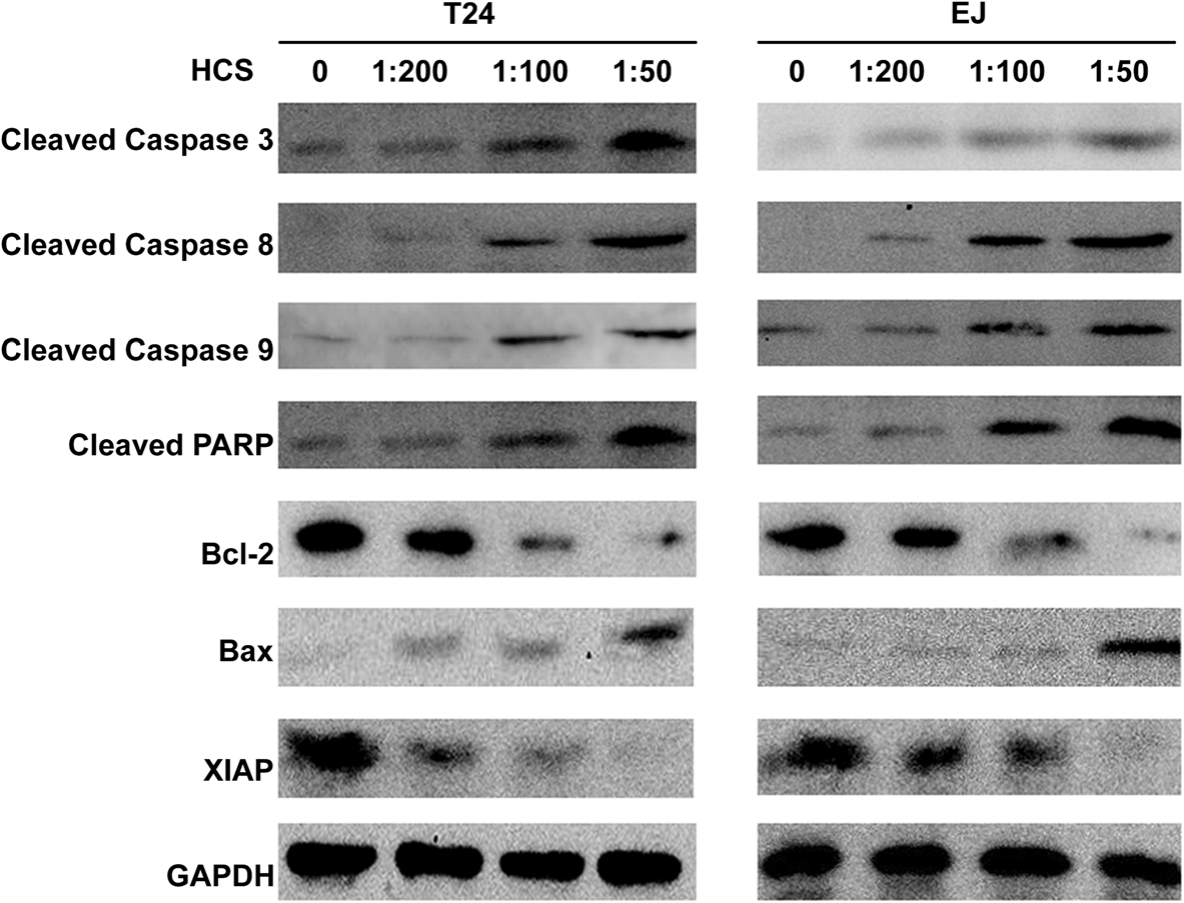
**Effect of HCS on the levels of apoptosis-related molecules in T24 and EJ cell lines.** The active (cleaved) forms of Caspase 3,8,9 and cleaved PARP were increased in a dose-dependent manner in HCS-treated T24 and EJ cells. the expression level of Bax was also elevated,whereas, the anti-apoptosis proteins such as Bcl-2 and XIAP were down regulated after HCS treatment.

To investigate the mechanism behind HCS-induced apoptosis, we detected the level of several apoptosis-related molecules by western blot. As is shown in Figure 2, the results revealed that the levels of the active (cleaved) forms of Caspase-3,-8,-9 and cleaved PARP were increased in a dose-dependent manner in HCS-treated T24 and EJ cells. We next determined whether HCS-induced apoptosis in bladder cancer cells is also associated with the modulation of the inhibitors of apoptosis proteins (IAPs) and Bcl-2 family proteins. Western blotting results indicated that HCS treatment decreased the expression of Bcl-2 and increased the expression of Bax. The expression of XIAP was also significantly decreased in both two cell lines after HCS treatment.

### HCS increases the expression of Fas, Fasl, TNF-α

**Figure 3:**
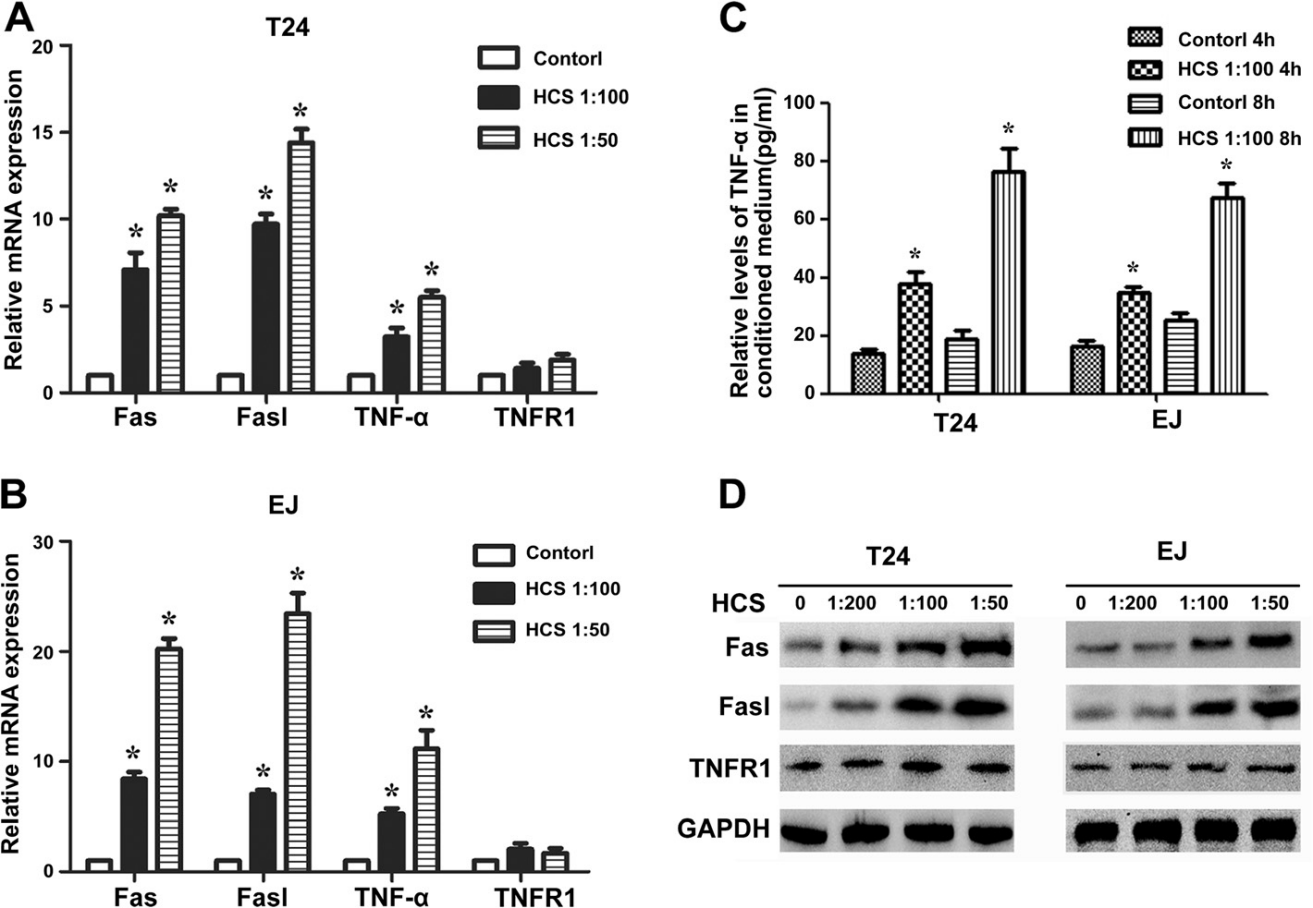
**HCS treatment upregulated the expression of Fas, Fasl and TNF-α.** The mRNA levels of Fas, Fasl, TNF-α and TNFR1 in T24 **(A)** and EJ **(B)** cells after HCS treatment. **(C)** Quantification of TNF-α in conditioned cell supernatant using ELISA. **(D)** The protein levels of Fas, Fasl and TNFR1 in T24 and EJ cells after HCS treatment were detected by western bolt. *, *P* < 0.05 vs control group.

**Figure 4:**
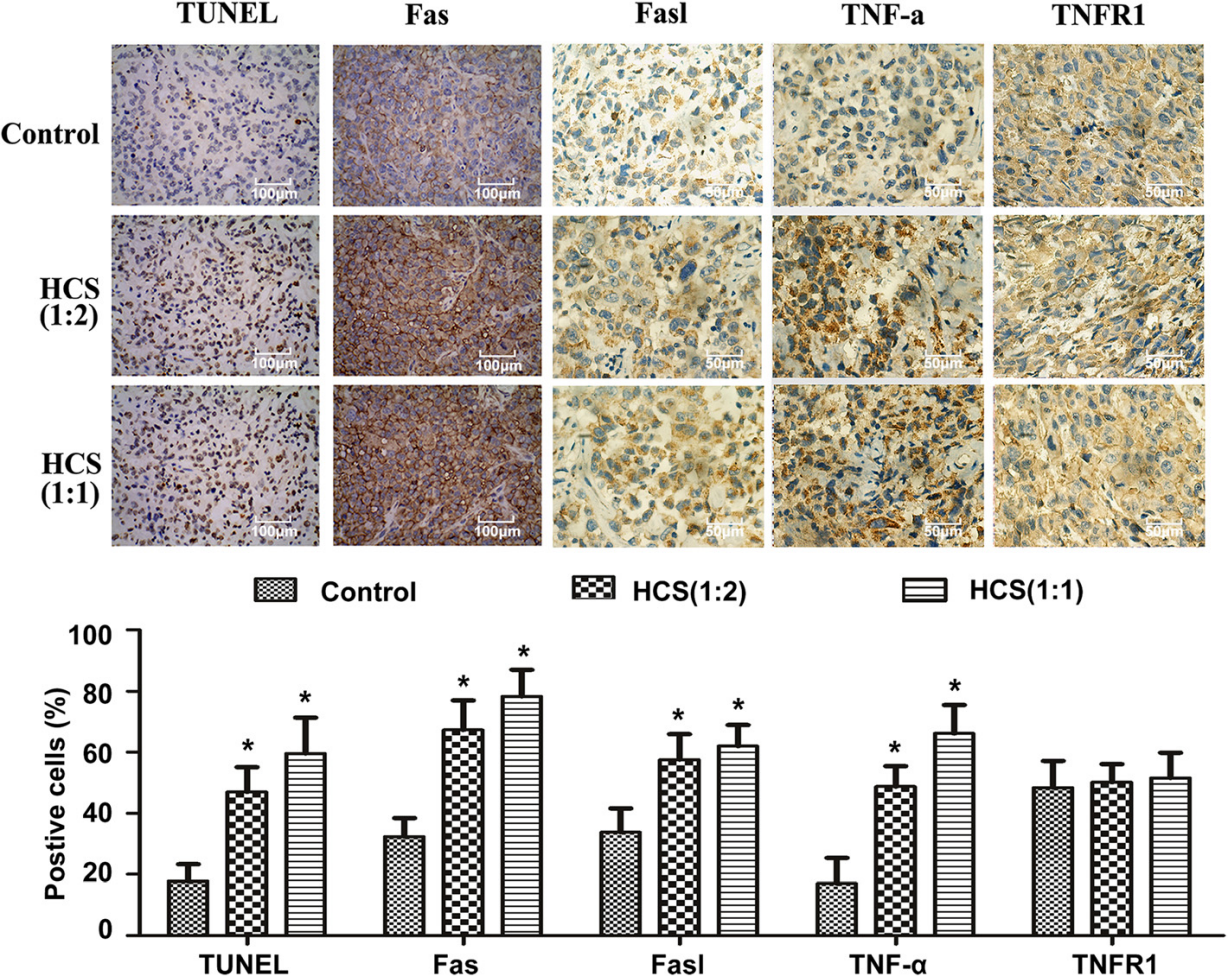
**HCS induced cell apoptosis and the expression of Fas, Fasl and TNF-α** ***in vivo*** **.** Representative results of the TUNEL staining of tumor sections and immunohistochemical analysis of Fas, Fasl, TNF-α and TNFR1 expression in control and HCS-treated T24 cell-transplanted mice. Columns are expressed as mean ± SD of five samples of each group. *, *P* < 0.05 compared to the control group.

To explore the underlying molecular signaling pathways by which HCS exerts antitumor effects, we examined the expression of Fas/Fasl and TNF-α/TNFR1 by PCR, western blot immunohistochemistry and ELISA. As is shown in Figure 3A, B, the mRNA levels of Fas, Fasl, TNF-α were increased in a dose-dependent manner in the two cell lines after HCS treatment. Furthermore, Western blotting results indicated that HCS treatment also increased the protein levels of Fas and Fasl, and ELISA results showed HCS could stimulate cells secrete TNF-α (Figure 3C, D). On the other hand, the expression of TNFR1 in both mRNA and protein levels had no changes after HCS treatment as shown in Figures 3 and 4.

### HCS induced cell death is mediated by Fasl/Fas and TNF-α/TNFR1 pathway

**Figure 5:**
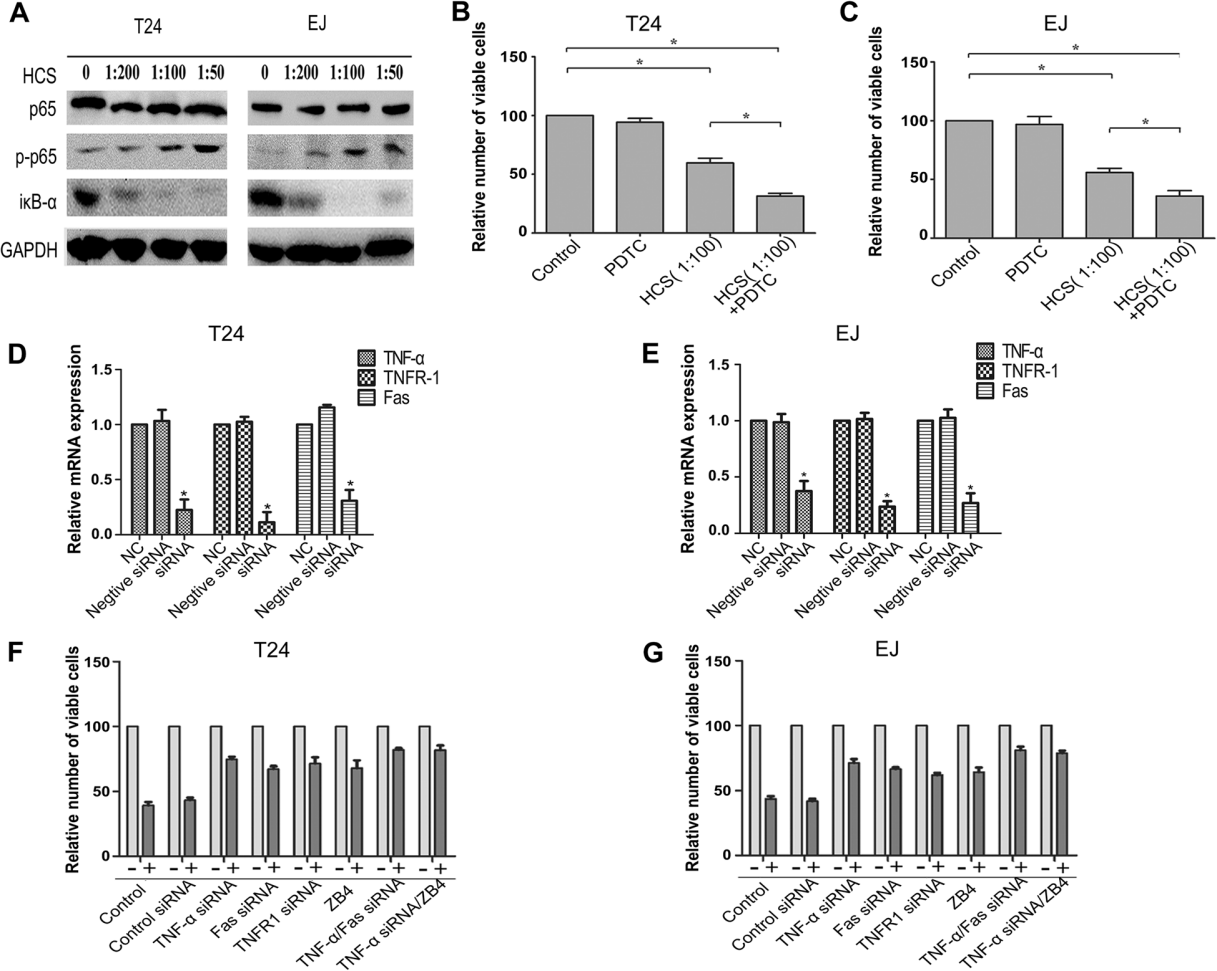
**The role of Fasl/Fas, TNF-α/TNFR1 and NF-κB pathway in HCS induced apoptosis. (A)** The protein levels of iκB-α , p65 and -p65 in T24 and EJ cells after HCS treatment were detected by western bolt. **(B, C)** Proliferation of cells treated with HCS(1:100) after inhibition of NF-κB pathway by PDTC. siRNA interference effects was tested through PCR in T24 and EJ cell lines **(D, E)**. Proliferation of T24 **(F)** and EJ **(G)** cells treated with HCS(1:100) after inhibition of Fasl/Fas and TNF-α/TNFR1 by siRNA or ZB4. *, *P* < 0.05.

To determine whether Fas/Fasl and TNF-α/TNFR1 expression is critical for the effect of HCS on bladder cancer cells, we knocked down TNF-a, TNFR1, Fas by siRNA or inhibited binding of FasL to its receptor by a blocking antibody (ZB4) prior to the addition of HCS, and then detected the cell viability. First, we confirmed that the siRNA effectively reduced the TNF-a, TNFR1, Fas mRNA level in T24 and EJ cells (Figure 5D, E). Then we found that TNF-a, TNFR1, Fas knockdown and the use of ZB4 significantly decreased the effect of HCS on cell proliferation compared with the control groups (Figure 5F, G). Furthermore, the effect of HCS on cell proliferation was more decreased by both of TNF-a/Fas siRNA and TNF-a siRNA/ZB4 treatment, which implicated both Fas/Fasl and TNF-α/TNFR1 as mediators of HCS-induced cell death.

### Activation of NF-κB pathway against HCS induce cell death

We also detected the activation of NF-κB pathway in T24 and RT-4 cells treated with HCS. Western blotting results showed HCS treatment decreased the expression of iκB-α and increased the expression of p-p65 in a dose-dependent manner, which indicated NF-κB pathway was activated by HCS (Figure 5A). To investigate whether NF-κB pathway got involved in HCS induced cell death, we tried inhibiting NF-κB activity with PDTC before HCS treatment, then we compared the cell viability with that of HCS treated alone. Interestingly, when we pretreated T24 and RT-4 cells with NF-κB inhibitor PDTC for 1 h before HCS treatment, the effect of HCS on the cell proliferation was strongly enhanced (Figure 5B, C). The results demonstrated that NF-κB pathway could attenuated the effect of HCS on bladder cancer cell growth.

### HCS inhibits human bladder tumor xenograft growth in athymic nude mice

**Figure 6:**
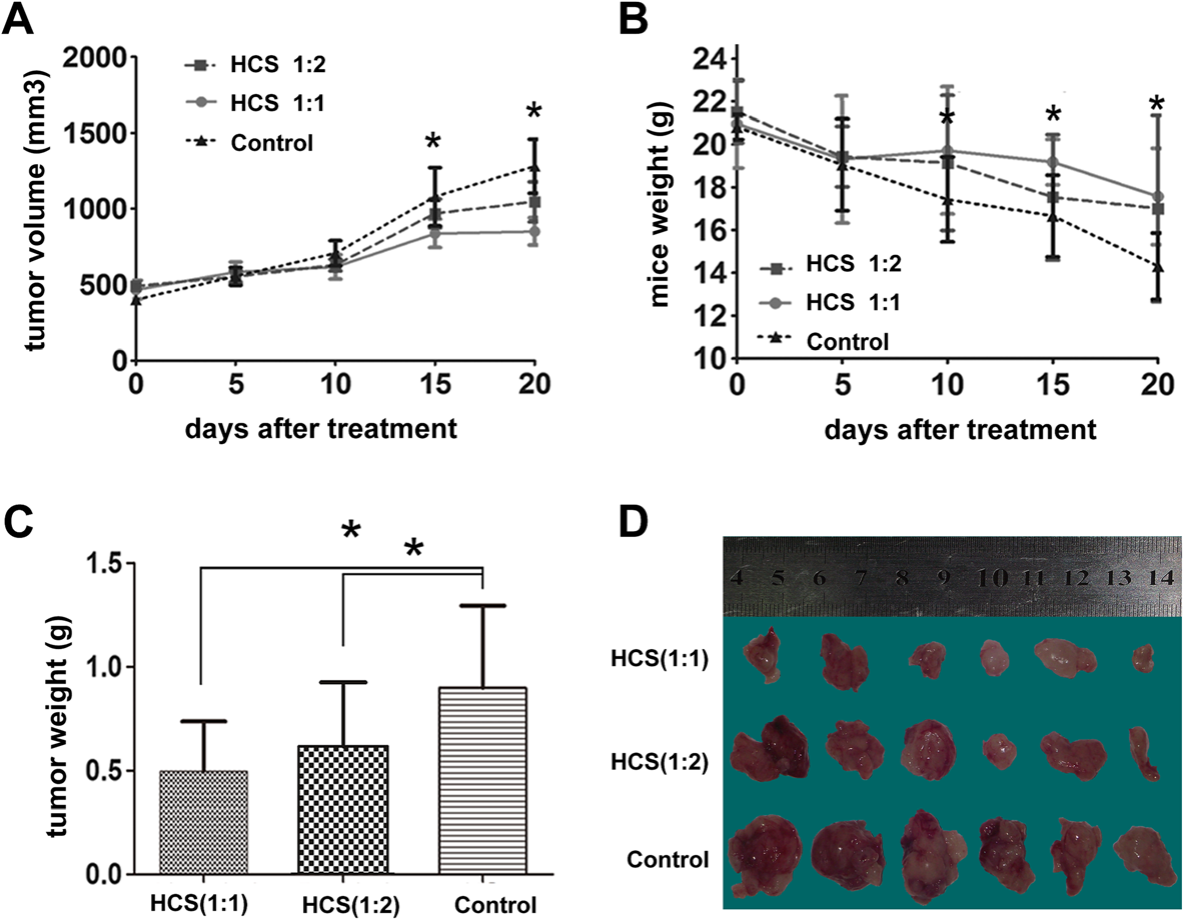
**HCS inhibited tumor growth** ***in vivo*** **.** Mean of tumor volume **(A)**, body weight of mice **(B)** and tumor weight **(C)** measured at the indicated number of days after mice were treated with HCS. **(D)** Representative pictures of tumor in control and HCS-treated T24 cell-transplanted mice. *, *P* < 0.05 compared to the control group.

We further investigated the effect of HCS on a transplanted tumor growth produced by T24 cells. Only two mice did not survive at the end of the experiment. As shown in Figure 6, the growth of T24 tumor xenografts was inhibited significantly following the injection of HCS at the dose levels of 1:2 and 1:1. The average bodyweight of the control group drops much more than the treatment groups (P < 0.05), and it’s speculated that the rapidly lose of bodyweight of the control mice may partially due to the progress of xenograft tumor.Besides, the average tumor masses in the control mice were nearly 2 fold (P < 0.05) greater than that of HCS-treated mice(1:1), while there is no significant difference between the two HCS treatment group (P > 0.05). The apoptosis of tumor cells was evaluated using TUNEL stain. The apoptotic cells were more prominent in the HCS-treated tumors than that of the control tumors as is seen in Figure 4. Then We detected the expression of Fas, Fasl, TNF-α and TNFR1 by IHC , and the increases in protein level of Fas, Fasl, TNF-α were also observed in the tumors of HCS-treated mice.

## Discussion

In recent years, the search for alternative anticancer agents in traditional medicine has been a potential strategy [16,17]. In this study, we explored that HCS induces apoptosis not only in cell culture, but also in tumor cells *in vivo*, as demonstrated in bladder cancer xenograft model treated with non-toxic dilutions of HCS.

To reveal the effect of HCS on bladder cancer cell lines, we tested the effect of HCS on cell growth and apoptosis. As revealed by MTS assay, HCS possessed an inhibitory ability on cell viability in a dose-dependent manner, while the inhibition is less remarkable in less malignant RT-4 and SV-HUC-1 cells. This phenomenon indicated that HCS may have less damage on normal bladder tissue. Furthermore, the morphologic changes induced by HCS indicate that cells were undergoing apoptosis. With annexin V/PI staining, HCS treatment significantly increased the proportion of apoptotic cells, which is increased with the rise of the concentration. These results confirmed that HCS exhibited an evident apoptosis-inducing effect on bladder cancer cells.

Caspases, a family of cysteine-aspartic proteases, play an essential role in apoptosis. Caspases are first synthesized as inactive pro-Caspases and regulated at a post-translational level. Activated Caspases eventually lead to apoptotic cell dismantling [18]. Poly (ADP-ribose) polymerase (PARP), a family of proteins involved in a variety of cellular processes including DNA repair and programmed cell death, is a downstream target of Caspase-3 [19,20]. Inhibitors of apoptosis proteins (IAPs) and Bcl-2 family proteins have also been shown to be important in the regulation of apoptosis [21,22]. We therefore tested the expression of Bax, Bcl-2, XIAP, cleaved PARP, cleaved Caspases 3, 8, 9, all are important members involved in apoptosis, through western blot after HCS treatment. The results confirmed that the apoptosis induced by HCS was related with the changes of these protein expression. So, what actually activated the Caspase cascade and initiated the apoptosis in bladder cancer cells treated with Huachansu? We tried to explore the result from both the intrinsic and extrinsic apoptosis pathway. The mitochondria-initiated intrinsic pathway, in which the release of cytochrome c from the mitochondrial matrix following loss of inner mitochondrial membrane integrity triggers formation of the apoptosome composed of Apaf-1, pro-Caspase-9, dATP, and cytochrome c [23]. The death receptor-initiated extrinsic pathway is another apoptosis process, in which death receptor ligation is followed by recruitment of adaptor molecules and activation of Caspase-8 or Caspase-10 [24,25]. We failed to detect the expression of cytochrome c by western blot for several times (data not shown), while the expression of Fas, Fasl, TNF-α were all elevated at both mRNA and protein level after HCS treatment. This results prompt us that the death receptor-initiated extrinsic pathway may contribute to the apoptosis induced by HCS in bladder cancer cells.

Fas is a member of the death receptor family, a subfamily of the tumor necrosis factor receptor superfamily. Binding to Fas by its physiological ligand, FasL, results in recruitment of adaptor molecules and activation of Caspase-8 or Caspase-10, to form death inducing signaling complex (DISC), transducing a downstream signal cascade resulting in apoptosis [26,27]. TNF-α is a pleiotropic ligand of tumor necrosis factor receptor 1 and 2 (TNFR1 and TNFR2) that can signal both cell survival and cell death [28,29]. The death-inducing complex assembles following internalization of the TNFR1 and consists of TNFR1-associated death domain protein(TRADD) and receptor-interacting protein kinase 1(RIPK1), which then recruit Fas-associated protein with death domain (FADD) and Caspase-8 to generate the DISC [30]. In this study, to determine whether TNF-a/TNFR1 and Fas/Fasl is required for HCS-induced cell death, we knocked down TNF-a, TNFR1, Fas by siRNA or inhibited binding of FasL to its receptor by a blocking antibody prior to the addition of HCS, then detected the cell viability. Importantly, downregulation of TNF-a, TNFR1, Fas or inhibition of Fas/Fasl interaction decreased the relative number of death cells induced by HCS, Figure 5 (F,G). Besides, TNF-a, TNFR1 and Fas knockdown with respective siRNA decreased the apoptosis rate induced by HCS in T24 cells, and the expression of cleaved caspase-3,-7,-8 and cleaved PARP also decreased as is shown in Additional file 1: Figure S1. Because of the above results we can conclude that HCS indeed affects bladder cancer cell growth which is mediated by TNF-a/TNFR1 and Fas/Fasl signaling pathway.

A major finding of this paper is that HCS not only reduced cell proliferation in cell culture but also inhibited tumor growth *in vivo*. We observed a significant increase in TUNEL-positive apoptotic cells in tumors from mice that were treated with HCS, and the protein level of Fas, Fasl, TNF-α in HCS-treated tumors were also up-regulated.

As we knew,the resistance of cells to TNF-α mediated apoptosisis attributed to TNF-α induced NF-κB activation [31], then we detected the activation of NF-κB pathway. As expected, NF-κB pathway was activated after HCS treatment. Then, we found inhibiton of NF-κB by the inhibitor could enhance the effect of HCS-induced cell death. The results indicated that the treatment of HCS plus chemotherapeutic drugs which could inhibit NF-κB pathway in bladder cancer may have better curative effect compared to monotherapy.

## Conclusions

Taken together, our study demonstrated that HCS induced-apoptosis in human bladder cancer was mediated by the activation Fas/Fasl and TNF-α/TNFR1. HCS may proved to be novel therapeutic strategy in the inhibition of carcinogenesis and progression of bladder cancer. Nevertheless, further studies are required to verify whether HCS could be used for an intravesical treatment.

## Additional file

Additional file 1:
**TNF-a, TNFR1 and Fas siRNA transfection inhibited apoptosis of T24 cell induced by HCS.** T24 cells are treated with TNF-a, TNFR1 and Fas siRNA for 48 h before HCS(1:100) treatment, And the apoptotic rate was detected by flow cytometry ,the expression of cleaved PARP, XIAP and cleaved caspase-3,-7,-8 was detected by western blot. Additional file 1: Figure S1 (A,B) TNF-a, TNFR1 and Fas knock down decreased the apoptosis rates of HCS induced apoptosis in T24 cell, Columns are expressed as mean ± SD of 3 independent experiments. *, *p* < 0.05 for HCS vs. control, #, p < 0.05 for positive siRNA vs. negative siRNA and control + HCS. HCS, huachansu (1:100). (C) The expression of cleaved caspase-3,-7,-8 and cleaved PARP was decreased whereas the expression of XIAP elevated compared to the negtive control after respective siRNA transfection. Lane 1, 2, 3, 4, 5, 6 is consistent with the group order of that in Figure S1 B.

## Abbreviations

HCS: Huachansu
TNF- a: Tumor necrosis factor- a
TNFR1: Tumor necrosis factor receptor 1
NF-κB: Nuclear factor kappa B
IAPs: Inhibitors of apoptosis proteins
