# Supplementary figures and images for: Huachansu suppresses human bladder cancer cell growth through the Fas/Fasl and TNF- alpha/TNFR1 pathway in vitro and in vivo
Source: J Exp Clin Cancer Res. 2015 Feb 25;34(1):21. doi: 10.1186/s13046-015-0134-9 (PMC4354737; doi:10.1186/s13046-015-0134-9)

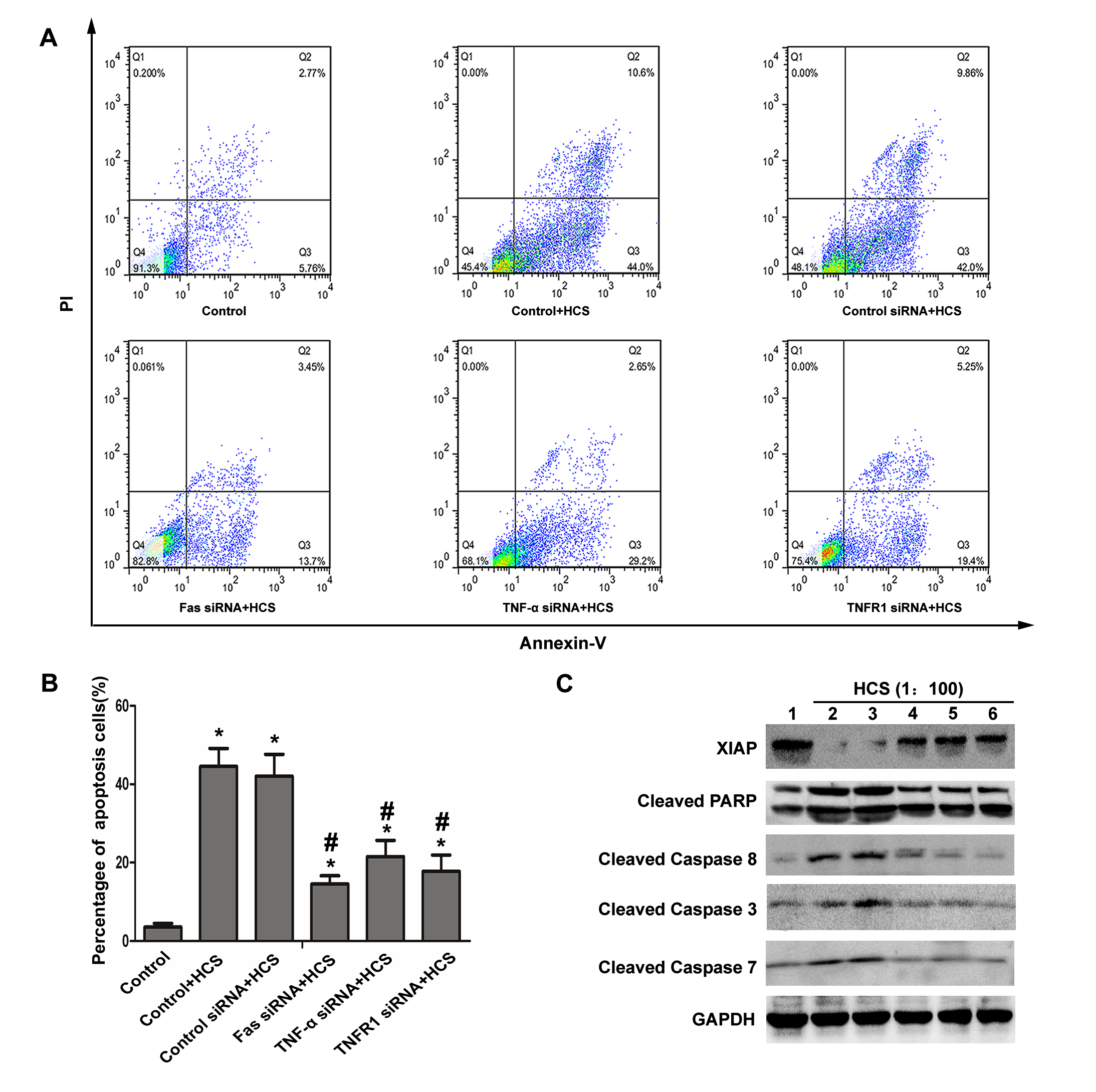

Supplement: Additional file 1: — TNF-a, TNFR1 and Fas siRNA transfection inhibited apoptosis of T24 cell induced by HCS. T24 cells are treated with TNF-a, TNFR1 and Fas siRNA for 48 h before HCS(1:100) treatment, And the apoptotic rate was detected by flow cytometry ,the expression of cleaved PARP, XIAP and cleaved caspase-3,-7,-8 was detected by western blot. Additional file 1: Figure S1 (A,B) TNF-a, TNFR1 and Fas knock down decreased the apoptosis rates of HCS induced apoptosis in T24 cell, Columns are expressed as mean ± SD of 3 independent experiments. *, p < 0.05 for HCS vs. control, #, p < 0.05 for positive siRNA vs. negative siRNA and control + HCS. HCS, huachansu (1:100). (C) The expression of cleaved caspase-3,-7,-8 and cleaved PARP was decreased whereas the expression of XIAP elevated compared to the negtive control after respective siRNA transfection. Lane 1, 2, 3, 4, 5, 6 is consistent with the group order of that in Figure S1 B. [file 13046_2015_134_MOESM1_ESM.png]
